# Clinical Parameters and Metabolomic Biomarkers That Predict Inhospital Outcomes in Patients With ST-Segment Elevated Myocardial Infarctions

**DOI:** 10.3389/fphys.2021.820240

**Published:** 2022-02-08

**Authors:** Jie Liu, Lei Huang, Xinrong Shi, Chungang Gu, Hongmin Xu, Shuye Liu

**Affiliations:** ^1^Clinical Laboratory Department, The Third Central Hospital of Tianjin, Tianjin, China; ^2^Tianjin Key Laboratory of Extracorporeal Life Support for Critical Diseases, Tianjin, China; ^3^Artificial Cell Engineering Technology Research Center, Tianjin, China; ^4^Tianjin Institute of Hepatobiliary Disease, Tianjin, China; ^5^Heart Center, The Third Central Hospital of Tianjin, Tianjin, China

**Keywords:** STEMI, mortality predictors, myocardial infarction, liquid chromatography-mass spectrometry, lipid metabolites

## Abstract

**Background:**

Postoperative risk stratification is challenging in patients with ST-segment elevation myocardial infarction (STEMI) who undergo percutaneous coronary intervention. This study aimed to characterize the metabolic fingerprints of patients with STEMI with different inhospital outcomes in the early stage of morbidity and to integrate the clinical baseline characteristics to develop a prognostic prediction model.

**Methods:**

Plasma samples were collected retrospectively from two propensity score-matched STEMI cohorts from May 6, 2020 to April 20, 2021. Cohort 1 consisted of 48 survivors and 48 non-survivors. Cohort 2 included 48 patients with unstable angina pectoris, 48 patients with STEMI, and 48 age- and sex-matched healthy controls. Metabolic profiling was generated based on ultra-performance liquid chromatography and a mass spectrometry platform. The comprehensive metabolomic data analysis was performed using MetaboAnalyst version 5.0. The hub metabolite biomarkers integrated into the model were tested using multivariate linear support vector machine (SVM) algorithms and a generalized estimating equation (GEE) model. Their predictive capabilities were evaluated using areas under the curve (AUCs) of receiver operating characteristic curves.

**Results:**

Metabonomic analysis from the two cohorts showed that patients with STEMI with different outcomes had significantly different clusters. Seven differentially expressed metabolites were identified as potential candidates for predicting inhospital outcomes based on the two cohorts, and their joint discriminative capabilities were robust using SVM (AUC = 0.998, 95% CI 0.983–1) and the univariate GEE model (AUC = 0.981, 95% CI 0.969–0.994). After integrating another six clinical variants, the predictive performance of the updated model improved further (AUC = 0.99, 95% CI 0.981–0.998).

**Conclusion:**

A survival prediction model integrating seven metabolites from non-targeted metabonomics and six clinical indicators may generate a powerful early survival prediction model for patients with STEMI. The validation of internal and external cohorts is required.

## Introduction

The popularity of primary percutaneous coronary intervention (PCI) and shorter reperfusion times has given rise to substantial number of patients with ST-segment elevation myocardial infarction (STEMI) who undergo timely coronary revascularization and obtain improved outcomes. However, the inhospital mortality rate remains substantial, ranging from 4 to 12% (Kristensen et al., 2014). To improve outcomes in this population, it is critical to stratify risk in the early stage of morbidity and administer intensive care to the high-risk groups.

Although extensive studies have been carried out regarding models predicting outcomes in patients with myocardial infarction, the vast majority of them are based on a single clinical or traditional biochemical index (Eitel et al., 2010; Zhang et al., 2018; Huang et al., 2019). Given that ischemia-reperfusion injury after STEMI involves multiple pathways and pathological processes, a multifactor prediction model integrating clinical and biochemical indexes might better stratify early risk after onset.

Metabonomics is an essential part of systems biology; it studies the metabolic changes of biological samples after stimulation, focusing on endogenous small molecules with a molecular weight less than 1,000 Da, including amino acids and lipids. The technology has enabled the measurement of thousands of metabolites, providing us with the metabolic fingerprint of an individual patient. In recent years, significant breakthroughs have been made in clarifying multiple cardiovascular disease phenotypes and regulatory mechanisms (Cheng et al., 2015; Onuh and Qiu, 2021; Surendran et al., 2021). Regarding coronary heart disease, the entire spectrum from non-obstructive coronary atherosclerosis to STEMI has been comprehensively characterized using metabonomics, and the established metabonomic-based biomarker models have been shown to predict outcomes at each stage accurately (Fan et al., 2016). Our previous study successfully characterized specific metabolic profiles in patients with STEMI under 45 years old (Huang et al., 2016) and in patients with STEMI with left main coronary artery disease (LMCAD, Huang et al., 2018) using a non-targeted metabolomic platform. Some important differential metabolites also proved to be valuable predictors of outcome.

In patients with STEMI, there is substantial interindividual heterogeneity in the complexity of the disease, resulting in fewer biomarkers that can be translated into clinical practice. Therefore, this study aimed to develop a non-targeted metabonomic-based survival prediction model for patients with STEMI using propensity score matching and generalized estimating equation (GEE) models.

## Materials and Methods

### Participant Selection

Our institute is a tertiary university hospital with a central laboratory accredited by the International Organization for Standardization and the College of American Pathologists (No. 8044075). The volume of primary PCI for STEMI is approximately 480 cases annually. The sample size was estimated using the MetaboAnalyst tool, based on our pilot dataset. Notably, 25 patients per group were shown to afford the study robustness (around 0.95) [we entered 200 as the maximum sample size per group and set the false discovery rate (FDR) cutoff value to 0.1].

We included two cohorts in this retrospective study. In Cohort 1, 48 successive patients who died in hospital (DSTEMI) and 48 propensity score-matched patients who survived to discharge (SSTEMI) were included from May 6, 2020 to April 20, 2021. The estimated propensity scores were calculated using a logistic regression model for inhospital survival as a function of the time from the onset of symptoms to entering into the hospital and the time of morbidity (day/night). These confounding factors can be a primary source of heterogeneity in metabolomic testing (Deidda et al., 2019). The matching was performed using a nearest-neighbor matching within 0.2 SDs of pooled propensity scores. In Cohort 2, 48 patients with unstable angina (UA) pectoris, 48 age- and sex-matched patients with STEMI, and 48 healthy controls were enrolled during the same period.

The diagnostic criteria of STEMI and UA referred to the *Third Universal Definition of Myocardial Infarction* (Thygesen et al., 2012) and *2019 ESC Guidelines for Diagnosing and Managing Chronic Coronary Syndromes* (Knuuti et al., 2020), respectively. Both were confirmed by subsequent coronary angiography. Patients with the following conditions were excluded: (1) non-atherosclerotic disease, (2) history of acute myocardial infarction (AMI), and (3) comorbidities including infection, liver or renal insufficiency, malignant tumor, and endocrine diseases.

### Plasma Collection and Ethics Statement

Plasma samples of all participants were collected immediately at admission. The blood samples that were anticoagulated with ethylenediaminetetraacetic acid were centrifuged at 3,000 rpm for 10 min at 4°C, and the resulting plasma was stored at –80°C.

All procedures involving human participants accorded with the ethical standards of the institutional and national research committee and with the 1964 Declaration of Helsinki and its later amendments or comparable ethical standards. The Bioethics Committee of the Third Central Hospital of Tianjin approved the study. Written informed consent was obtained from all patients.

### Quality Control Analysis

The performance of quality control (QC) solution in the entire sample analysis process was an assessment of the stability of the ultra-performance liquid chromatography and mass spectrometry (UPLC/MS) system. The QC sample solution was prepared using a mixture of equal aliquots from each sample. Ten consecutive QC samples were injected before the test samples were run, and the remaining QC samples were inserted into the sequence after every ten samples were analyzed. The sample sequence was randomly generated using the random number generator in Microsoft Excel, and cross-contamination was obviated by inserting a blank between adjacent samples. The system reached equilibrium after the first ten control samples and remained stable during the sample analysis process. The QC standard was set as follows: (1) ion peaks were defined as reliable peaks when their intensity was in the range of ± 30% average ion intensity; (2) a QC sample was qualified if 70% ion peaks were reliable; and (3) the experimental data were accepted only when 60% QC was qualified (Dunn et al., 2008).

### Critical Metabolite Analysis and Data Processing

Thermo Fisher Scientific provided a UPLC/MS system (Thermo Fisher Scientific, Franklin, MA, United States). The chromatographic column was a Thermo Hypersil GOLD reversed-phase C18 (2.1 mm I.D. × 50 mm, 1.9 μm). The MS was performed on the LTQ Orbitrap XL system (Thermo) equipped with an electrospray ionization ion source.

The sequence of the sample analysis was randomly generated using Microsoft Excel. The data obtained from the UPLC-LTQ Orbitrap XL platform analysis were directly imported into MZmine 2.0 software for data preprocessing (Katajamaa and Oresic, 2005), including peak detection, comparison, and standardization (taking the total ionic strength of each sample as the standardization factor). The 80% rule was used to remove the variable with too many missing values (Sabatine et al., 2005), and MZmine was used to assign values to the variables with fewer missing values. The data table obtained from the analysis was a two-dimensional peak table (I × J). Each row (I) represents a sample, each column (J) represents a parameter, i.e., m/z, and the numerical value represents the ion peak intensity (peak integral area). The data were imported to SIMCA-P + 12.0.1.0 software (Umetrics, Umeå, Sweden) to construct the disease-distinguishing models of the principal component analysis (PCA) and the orthogonal partial least squares discriminant analysis (OPLS-DA) (Zhang et al., 2019).

The variable influence on projection (VIP) score was calculated based on the partial least squares (PLS) weights. The variables with VIP > 1.0 were deemed relevant for group discrimination. VIP statistics and S-plot were used to obtain the significant variables for subsequent metabolism pathway analysis. The selected metabolites were then preliminarily confirmed. The supervised model was validated. The permutation test was used to confirm no overfitting in the study when the intercept of R2 in the Y-axis was less than 0.4 and that of Q2 in the Y-axis was less than 0.05. Then, we searched Mass Frontier 6.0, Kyoto Encyclopedia of Genes and Genomes,^1^ and Human Metabolome Database^2^ using the exact number (m/z) mass and MS/MS spectra to identify the selected critical metabolites (Chen et al., 2008).

### Statistical Analysis

IBM SPSS Statistics for Windows software package, version 25 (IBM Corp., Armonk, NY, United States) and SIMCA-P + 12.0.1.0 software (Umetrics, Umeå, Sweden) were used for statistical analysis. Metric data were expressed as mean values with SD or median values with interquartile ranges. The qualitative data were expressed as frequency and composition. The metabolites were screened using the Mann–Whitney *U* test. The comprehensive metabolomic data analysis was performed using MetaboAnalyst version 5.0 (Pang et al., 2021).

The GEE is a helpful algorithm model to estimate binary outcomes (survival inhospital yes/no) when the clustered data are used (different lesion vessels of one patient). As all clinical data were presented and analyzed at the site level, subject-level parameters such as plasma biochemistry were presented in an adjusted form and assigned to the vessels with stenosis in the patient. Quasi-likelihood under the independence model criterion (QIC) was calculated, and the model with the smallest QIC was more parsimonious (Pan, 2001). The discrimination ability was assessed using the receiver operating characteristic (ROC) curve analyses. All tests were two-sided; the significance level was set at 0.05.

## Results

### Comparison of Baseline Characteristics Between Groups

As shown in Table 1, in Cohort 1 (UA vs. STEMI), the STEMI group had a significantly higher percentage of neutrophils and B-type natriuretic peptides. In Cohort 2 [patient with STEMI pairs with different outcomes after propensity score methods (PSMs)], compared with the survival group, the death group was older and had a worse cardiac function, a higher proportion of neutrophils and peak myocardial enzymes, and a higher proportion use of mechanical circulatory assistance devices.

**TABLE 1:** Comparison of baseline characteristics.

Parameters	Cohort 1			Cohort 2		
	UA (*n* = 48)	STEMI (*n* = 48)	P	SSTEMI (*n* = 48)	DSTEMI (*n* = 48)	P
Age	61.79 ± 7.77	64.88 ± 11.81	0.134	63.02 ± 9.94	74.94 ± 10.94	< 0.01
Male n (%)	26(54.17%)	31(64.58%)	0.299	33(68.75%)	26(54.17%)	0.142
S2D(hours)	—	2.6(2.2,4.8)	—	2.00(2.00,4.00)	4.00(2.00,5.00)	0.261
Hypertension n (%)	34(70.83%)	32(66.67%)	0.660	30(62.50%)	34(70.83%)	0.386
Diabetes n (%)	19(39.58%)	17(35.42%)	0.673	19(39.58%)	16(33.33%)	0.525
Heart rate (bpm)	76.10 ± 10.74	78.38 ± 15.94	0.415	77 ± 14.93	87.29 ± 19.60	0.005
SBP (mmHg)	140.69 ± 16.31	130.19 ± 24.65	0.016	131.35 ± 23.75	108.06 ± 23.02	< 0.01
Emergency PCI, n(%)	—	34(70.83%)		38(79.16%)	29(60.42%)	0.045
IABP or ECMO use	0(0)	3(6.3%)	0.242	3(6.25%)	17(35.42%)	< 0.01
Culprit vessel n (%)			0.897			0.774
LAD	18(37.50%)	21(43.75%)		27(56.25%)	30(62.50%)	
Lcx	4(8.33%)	3(6.25%)		4(8.33%)	2(4.17%)	
LM	3(6.25%)	2(4.17%)		5(10.42%)	6(12.50%)	
RCA	23(47.92%)	22(45.83%)		12(25.00%)	10(20.83%)	
Vessels involved No.	2.00(2.00,3.00)	3.00(2.00,3.00)	0.818	3.00(2.00,3.00)	3.00(2.00,3.00)	0.28
LVEF	57.54 ± 1.90	49.29 ± 5.05	< 0.01	49.33 ± 4.72	44.06 ± 8.73	< 0.01
BNP (pg/ml)	25.45(21.95,43.75)	60.50(19.48,205.38)	0.001	49.10(19.23,205.38)	109.00(29.30,219.38)	0.668
CKMB peak value (U/L)	15.00(12.00,20.75)	3.30(0.83,14.50)	0.288	2.9(0.70,10.30)	6.55(1.98,24.50)	0.014
Neutrophils%	66.39 ± 9.16	72.46 ± 10.43	0.003	72.25 ± 10.55	77.67 ± 11.16	0.016
TG(mmol/L)	1.26(1.04,1.95)	1.32(0.95,1.91)	0.987	1.23(0.93,1.91)	1.41(0.99,2.01)	0.831
TC(mmol/L)	4.26 ± 0.99	4.55 ± 0.73	0.106	3.87 ± 1.24	4.30 ± 0.92	0.058

*UA, uric acid; LDH, lactate dehydrogenase; CK, creatine kinase isoenzyme; CKMB, creatine kinase isoenzyme-MB; TG, triglyceride; TC, total cholesterol; HDL, high-density lipoprotein; LDL, low-density lipoprotein; S2D, symptom to door; LVEF, left ventricular ejection fraction.*

### Discriminant Analyses to Patients With ST-Segment Elevation Myocardial Infarction With Different Outcomes

**FIGURE 1:**
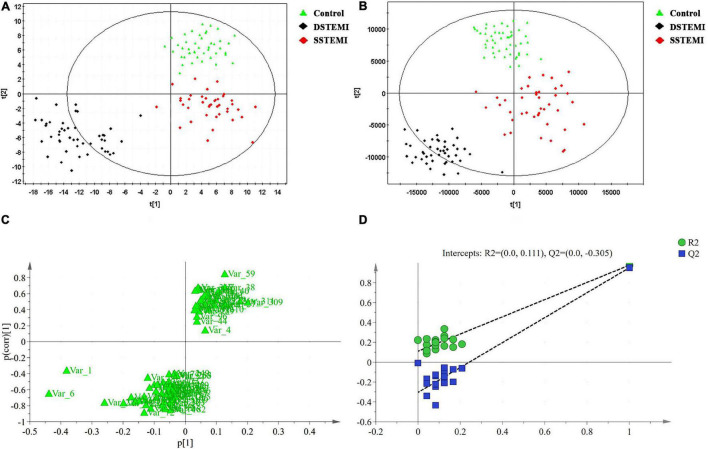
Discriminant analyses and validation. The principal component analysis (PCA) model **(A)** is established with seven principal components for SSTEMI, DSTEMI, and control group. The orthogonal partial least squares discriminant analysis (OPLS-DA) model is used to magnify the nuances **(B)**. S-plot **(C)** and the supervised model **(D)** are validated. DSTEMI, death group of ST-segment elevation myocardial infarction; SSTEMI, survival group of ST-segment elevation myocardial infarction.

QC samples that were inserted into the test samples were qualified, and the qualified ratio was 83.3%, which means that the analytical results are reliable. The PCA model (Figure 1A) was established using seven principal components (R2X = 40.2%, Q2 = 13.9%) for SSTEMI, DSTEMI, and control groups. The control, SSTEMI, and DSTEMI groups displayed a tendency to distinguish from one another, suggesting a trend of intergroup separation on the score plots. An OPLS-DA model was then used to magnify the nuances. The OPLS-DA model (Figure 1B) contained two main predictive ingredients and five orthogonal principal components (R2X = 79%, R2Y = 83.2%, Q2 = 65%). R2X and R2Y represent the interpretation rate of the model to the X and Y matrix, respectively. Q2 indicates the prediction ability of the model. The closer their values are to 1, the better the fitting degree of the model is, and the more accurate the training set samples can be divided into their original attribution. This model demonstrated good performance and predictive ability. Compared to the S-plot (Figure 1C), the OPLS-DA model found the same important metabolites using S-plot. The supervised model was validated using the permutation test; R2 and Q2 are shown in Figure 1D. R2 was 0.111, and Q2 was -0.305, suggesting no overfitting.

**FIGURE 2:**
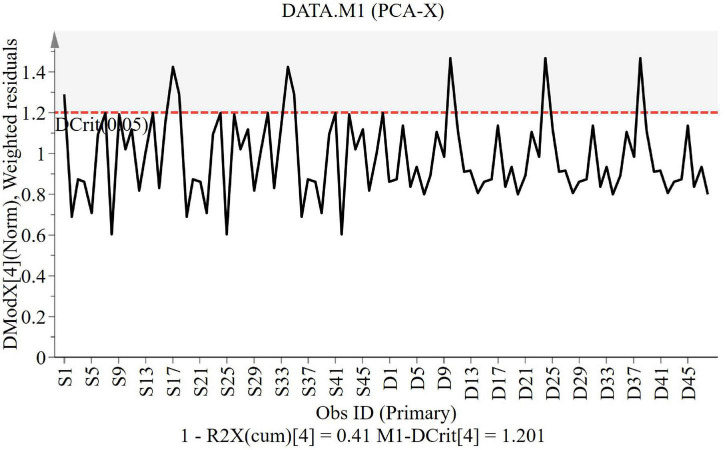
DModX line of the study groups. PCA, principal component analysis.

The DModX line plot (Figure 2) is used to evaluate the presence of outliers. Outliers are more than two times the given red dashed threshold. It can be found that there are no outliers in this study.

### Selection of Metabolites, Metabolism Pathways, and Functional Analysis

**FIGURE 3:**
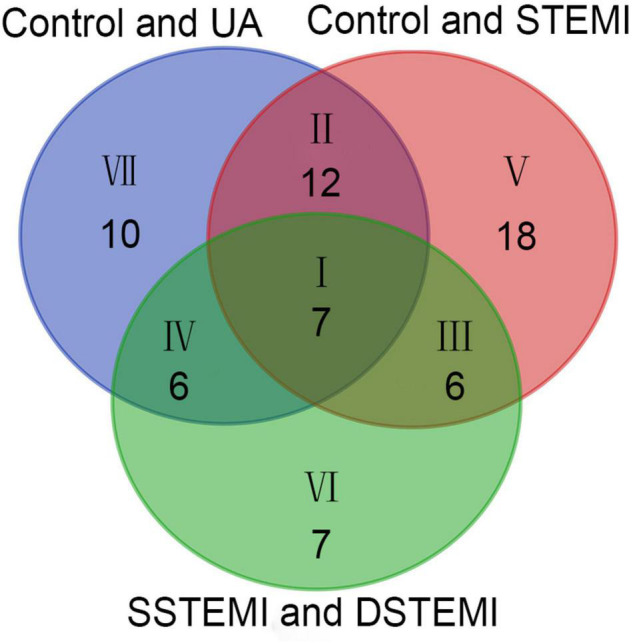
Venn diagram of the metabolites identified from control vs. UA, control vs. STEMI, and SSTEMI vs. DSTEMI. DSTEMI, death group of ST-segment elevation myocardial infarction; SSTEMI, survival group of ST-segment elevation myocardial infarction; UA, unstable angina.

The key metabolites relating to Cohorts 1 and 2, which affect the clustering tendency within groups, were selected, and the ions, including zero in the CI in the VIP diagram or the coefficient plot, were excluded (Sabatine et al., 2005). Following these steps, 85 different metabolites were identified from the extracted 362 metabolites using the non-parametric test. After the Mann–Whitney *U* test, 35, 43, and 26 metabolites were identified from comparing the control and UA groups, control and STEMI groups, and SSTEMI and DSTEMI groups (Figure 3 and Supplementary Table 1). The common part of the three groups of the comparison, i.e., seven metabolites, was closely associated with the onset of STEMI and different outcomes (Table 2).

**TABLE 2:** Identification of seven metabolites.

ID	m/z	RT	Metabolite	HMDB	KEGG	PubChem	UA vs Ctrl	STEMI vs UA	DSTEMI vs SSTEMI
Var22	546.35	8.59	LysoPC(18:0)	HMDB0010384	C04230	497299	Down	Down	Down
Var310	318.24	7.29	9-cis-Retinoic acid	HMDB0002369	C15493	449171	Up	Up	Up
Var214	338.27	6.31	Dehydrophytosphingosine	-	-	-	Up	Up	Up
Var107	499.29	4.31	N-Acetyl-leukotriene E4	HMDB0005084	C11361	53477792	Down	Down	Down
Var323	358.37	7.94	4-Hydroxy-6-docosanone	HMDB0035667	-	131751834	Down	Down	Down
Var40	103.05	1.16	3-Methyl-2-butene-1-thiol	HMDB0031529	-	146586	Up	Up	Up
Var38	120.08	1.16	L-Threonine	HMDB0000167	C00188	6288	Up	Up	Up

*DSTEMI, deaths of ST-segment elevation myocardial infarction; RT, retention time; SSTEMI, survivors of ST-segment elevation myocardial infarction; UA, unstable angina.*

**FIGURE 4:**
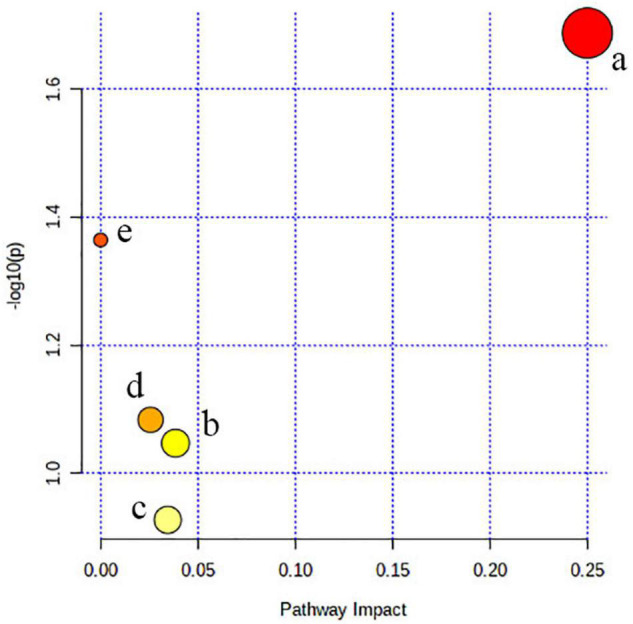
Pathway analysis. All the matched pathways are shown as circles. (a) Valine, leucine, and isoleucine biosynthesis, (b) glycerophospholipid metabolism, (c) aminoacyl-tRNA biosynthesis, (d) glycine, serine, and threonine metabolism, and (e) retinol metabolism.

The metabolism pathway analysis showed that the seven metabolites were responsible for the metabolism of retinol, glycine, serine, threonine, and glycerophospholipid and the biosynthesis of valine, leucine, isoleucine, and aminoacyl-tRNA (Figure 4).

### Biomarker Identification and Performance Evaluation

**FIGURE 5:**
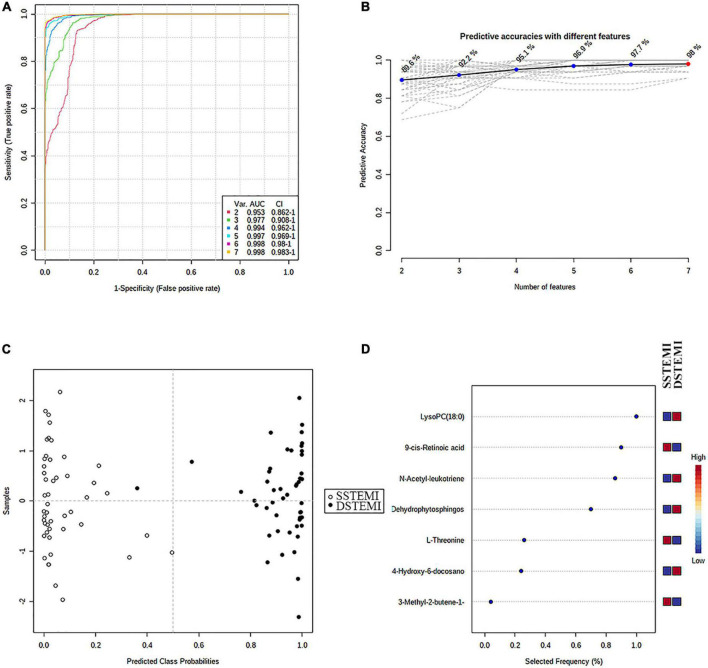
The process of feature selection, model building, and performance evaluation performed multiple times through the Monte-Carlo cross-validation *via* MetaboAnalyst 5.0 **(A)** ROC curves of all models based on the cross-validation performance. Model 6 had the largest AUC and **(B)** the highest predictive accuracy. One DSTEMI sample goes into SSTEMI samples in predicted class probabilities **(C)**. Seven significant features ranked based on their frequencies of being selected **(D)** during cross-validation.

Then, the linear support vector machine (SVM) for classification and “SVM built-in” for the feature ranking method was generated as a multivariate algorithm to perform biomarker identification. The ROC curves from all models based on the cross-validation performance indicated that Model 6 had the largest AUC (0.998) and highest predictive accuracy (98.0%) (Figures 5A,B). One DSTEMI sample went into SSTEMI samples in predicted class probabilities, suggesting that the false positive rate of the model was 2.08% (1/48) (Figure 5C). In the inhospital outcome predicting, model building, and performance evaluation based on the Monte Carlo cross-validation, lysophosphatidylcholine (LysoPC) (18:0) had the greatest chance to appear in the predictive biomarker panel with the highest frequency (Figure 5D), suggesting that it is the most important biomarker in the multiple biomarker panel. The corresponding cutoff values with associated sensitivity and specificity is shown in Table 3.

**TABLE 3:** Receiver operating characteristic (ROC) curve analysis for individual biomarkers.

Metabolite	AUC	*T*-tests	Log2 FC	Cutoff	Sensitivity	Specificity	LR +	LR-
L-Threonine	0.9149(0.862–0.964)	1.1775E-13	–0.5072	6760000	0.7083	0.9792	34.0	0.2979
3-Methyl-2-butene-1-thiol	0.8594(0.768–0.922)	2.0004E-8	–0.4731	5780000	0.625	0.9792	30.0	0.3830
N-Acetyl-leukotriene E4	0.9523(0.909–0.98)	1.836E-21	1.9233	501000	0.9792	0.7917	4.7	0.0263
4-Hydroxy-6-docosanone	0.8889(0.829–0.943)	2.0327E-10	0.5019	535000	0.6667	0.9792	32.0	0.3404
LysoPC(18:0)	0.9566(0.921–0.983)	6.3462E-18	0.6343	17900000	0.98	0.8	4.7	0.0263
Dehydrophytosphingosine	0.9267(0.873–0.969)	1.0436E-10	–3.779	270000	0.7292	0.9792	35.0	0.2766
9-cis-Retinoic acid	0.9557(0.916–0.985)	0.001197	–8.4275	101.865	0.875	0.875	7.0	0.1429

*AUC, area under the curve; FC, fold change; LR, likelihood ratio.*

### Univariate Generalized Estimating Equation Model Analysis of Inhospital Outcome in Patients With ST-Segment Elevation Myocardial Infarction

In the univariate GEE model, a total of 246 lesion vessels were included to predict inhospital outcomes in patients with STEMI; 118 lesion vessels survived, and 128 lesion vessels did not. The influence of these parameters on the inhospital outcome is shown in Tables 4, 5. As shown in Table 5, (1) increasing age per year, intra-aortic balloon pump (IABP)/extracorporeal membrane oxygenation (ECMO) use, no emergency PCI, and faster heart rate, (2) a higher percentage of neutrophils, higher levels of total cholesterol, L-threonine, 3-methyl-2-butene-1-thiol, dehydrophytosphingosine, 9-*cis*-retinoic acid, and (3) lower levels of systolic pressure, left ventricular ejection fraction, *N*-acetyl-leukotriene E4, LysoPC (18:0), and 4-hydroxy-6-docosanone were significantly correlated to a higher probability of inhospital mortality, which is confirmed by the Monte Carlo cross-validation results.

**TABLE 4:** Relationships between baseline parameters (clinical parameters combined with biomarkers) and inhospital outcome of patients with ST-segment elevation myocardial infarction (STEMI) in univariate generalized estimating equation (GEE) models.

Parameters	Survival inhospital				
	Yes	No	Wald Chi-Square	P	QIC
	118	128			
Age (years)	68.23 ± 0.93	74.87 ± 0.99	9.293	0.002	292.756
Sex: Male/Female	79/39	72/56	1.018	0.31	349.246
Hypertension, n (%)	80 (67.80)	89 (69.53)	0.03	0.86	352.094
Diabetes, n (%)	44 (37.29)	43 (33.59)	0.129	0.72	351.73
S2D (hours)	2.5 (2.00,5.00)	4.00 (2.00,5.00)	3.779	0.63	351.859
Heart rate (bpm)	76.55 ± 14.41	85.67 ± 19.94	4.106	0.04	337.404
SBP (mmHg)	130.2 ± 24.17	108.24 ± 23.05	17.228	< 0.01	302.87
Emergency PCI, n (%)	95 (80.51)	76 (59.38)	4.369	0.04	339.008
CPR before reperfusion, n (%)	7 (5.93)	8 (6.25)	0.004	0.95	352.306
IABP or ECMO use	10 (8.47)	46 (35.94)	6.947	0.01	324.813
LVEF (%)	49.2 ± 4.58	44.63 ± 8.12	11.503	0.001	321.085
BNP (pg/ml)	63.5 (26.15,224.00)	108 (26.5,211.52)	0.102	0.75	351.41
CKMB (ng/ml)	2.9 (0.7,10.53)	5.6 (2.2,24.5)	3.783	0.05	334.018
Neutrophils%	70.91 ± 10.06	77.66 ± 11.21	6.204	0.01	329.642
TG (mmol/l)	1.23 (0.92,1.92)	1.39 (0.97,1.87)	0.004	0.95	352.009
TC (mmol/l)	3.85 ± 1.15	4.31 ± 0.87	4.298	0.04	339.157
L-Threonine	48.85 (43.43, 61.11)^a^	70.43 (58.12, 83.08)^a^	61.37	< 0.01	199.31
3-Methyl-2-butene-1-thiol	39.32 (36.74, 52.71)^a^	59.05 (45.18, 65.67)^a^	42.26	< 0.01	253.04
N-Acetyl-leukotriene E4	60.93 (50.95, 65.99)^b^	0.44 (0.17, 36.40)^b^	57.81	< 0.01	149.20
4-Hydroxy-6-docosanone	6.90 (5.71, 7.26)^a^	5.23 (4.37, 5.91)^a^	60.54	< 0.01	202.37
LysoPC (18:0)	200.00 (181.60, 217.85)^a^	136.33 (101.83, 155.47)^a^	54.79	< 0.01	145.35
Dehydrophytosphingosine	0.60 (0.35, 3.29)^b^	38.55 (2.33, 74.87)^b^	29.36	< 0.01	204.36
9-cis-Retinoic acid	59.63 (45.90, 84.96)	1122.53 (227.01,24878.4)	8.97	< 0.01	306.59

a: *105; b: *104.

*BNP, B-type natriuretic peptide; CKMB, creatine kinase isoenzymes; CPR, cardiopulmonary resuscitation; ECMO, extracorporeal membrane oxygenation; IABP, intra-aortic balloon pump; LVEF, left ventricular ejection fraction; LysoPC, lysophosphatidylcholine; PCI, percutaneous coronary intervention; QIC, quasi-likelihood under the independence model criterion; SBP, systolic pressure; S2D, symptom to door; TC, total cholesterol; TG, triglyceride.*

**TABLE 5:** Univariate GEE model for patients with ST-segment elevation myocardial infarction (STEMI) with the full dataset for mortality.

Predictors	OR (95% CI)	p	QIC	AUC	Cut-off	Sensitivity	Specificity
**Clinical Parameters**							
Age (years)	1.105(1.036,1.179)	0.002	292.756	0.829(0.819,0.838)	> 74.500	0.679	0.871
Heart rate (bpm)	1.03(1.001,1.061)	0.04	337.404	0.687(0.677,0.697)	> 76.000	0.836	0.614
SBP (mmHg)	0.962(0.944,0.98)	< 0.01	302.870	0.742(0.682,0.802)	< 99.500	0.941	0.437
Emergency PCI, n(%)	0.354(0.134,0.937)	0.04	339.008	0.606(0.535,0.676)	–	–	–
IABP or ECMO use	6.059(1.587,23.13)	0.01	324.813	0.631(0.618,0.644)	–	–	–
LVEF (%)	0.889(0.831,0.952)	<0.01	321.085	0.644(0.575,0.713)	< 49.500	0.500	0.742
Neutrophils%	1.06(1.013,1.111)	0.01	329.642	0.688(0.679,0.698)	> 74.750	0.660	0.673
TC(mmol/l)	1.572(1.025,2.412)	0.04	339.157	0.62(0.605,0.634)	> 3.715	0.833	0.478
**Metabolites**							
L-Threonine	1.016(1.012,1.021)	< 0.01	199.31	0.915(0.862–0.964)	6760000	0.708	0.979
3-Methyl-2-butene-1-thiol	1.012(1.009,1.016)	< 0.01	253.04	0.859(0.768–0.922)	5780000	0.625	0.979
N-Acetyl-leukotriene E4	0.880(0.851,0.909)	< 0.01	149.20	0.952(0.909–0.98)	501000	0.979	0.792
LysoPC(18:0)	0.991(0.989,0.993)	< 0.01	145.35	0.957(0.921–0.983)	17900000	0.98	0.8
Dehydrophytosphingosine	1.101(1.063,1.14)	< 0.01	204.36	0.927(0.873–0.969)	270000	0.729	0.979
4-Hydroxy-6-docosanone(*10^5^)	0.808(0.765,0.852)	< 0.01	202.37	0.889(0.829–0.943)	535000	0.667	0.979
9-cis-Retinoic acid(*10^4^)	1.065(1.022,1.110)	< 0.01	306.59	0.956(0.916–0.985)	101.865	0.875	0.875

*AUC, area under the curve; ECMO, extracorporeal membrane oxygenation; GEE, generalized estimating equation; IABP, intra-aortic balloon pump; LVEF, left ventricular ejection fraction; LysoPC, lysophosphatidylcholine; OR, odds ratio; PCI, percutaneous coronary intervention; QIC, quasi-likelihood under the independence model criterion; SBP, systolic pressure; TC, total cholesterol.*

The ROC curve analyses were then performed to evaluate the discrimination performance of the GEE model on the inhospital outcome. A model comprised of seven hub metabolites had a robust performance (AUC = 0.981, 95% CI 0.969–0.994). The discrimination capability improved after combining clinical factors (AUC = 0.99, 95% CI 0.981–0.998).

## Discussion

This study was initially designed to identify biomarker signatures to predict the short-term outcomes of patients with STEMI; we established a composite predictive model comprised of seven metabolites and six traditional clinical variants. Our findings revealed that the combined survival predictive model had excellent discriminative power for the inhospital outcomes of patients with STEMI.

This study introduced some innovations. First, patients with UA (UA vs. STEMI) and patients with STEMI with different outcomes were included. As a preceding form, UA has a similar pathophysiological basis to STEMI. We were more likely to obtain early specific biomarkers for the outcome by selecting the intersection of differential metabolites of the two cohorts. Second, STEMI is often associated with multiple-vessel involvement, and the non-culprit vessels with stenosis greater than 50% were also closely related to the outcome (Engstrøm et al., 2015; Smits et al., 2017). Thus, all involved vessels in each patient would be used as objects of analysis. Because the sites of diseased vessels within a patient could be more correlated to one another than sites between patients, a GEE model was used to determine the associations between outcome and the clustered parameters at baseline. In fact, the metabolite panel with the most parsimony robustly predicted the outcomes in patients with STEMI.

Sampling times vary widely in the previous studies. In these studies, blood collection times included overnight fasting, immediately after admission (non-fasting), and at different time points after admission. The ideal sampling time should be when the metabolic state is stable. However, patients with STEMI are always in a state of metabolic deviation, so there is no stable metabolic state for them. Under these circumstances, we used PSM to eliminate the heterogeneity between groups in the time interval from morbidity to sampling and the time of STEMI onset to improve the accuracy of differential metabolite screening.

A quailed biomarker should meet the following prerequisites, including accurate and valid measurement results and clinical benefits to patients. Due to the interindividual variability and disease complexity, few biomarker candidates have been translated into clinical practice in STEMI, requiring further research. The metabolomic-based studies in acute coronary syndrome have already demonstrated immense potential for biomarker discovery and mechanistic insights by identifying metabolomic signatures (e.g., branched-chain amino acids, succinate, and hydrogen sulfide) associated with disease progression or poorer outcomes (Ali et al., 2016; Huang et al., 2016; Du et al., 2018; Kohlhauer et al., 2018).

Among the seven metabolites identified in this study, LysoPC (18:0), 9-*cis*-retinoic acid (9cRA), and *N*-acetyl-leukotriene E4 occupied the first three places in terms of the weight of the prediction. Consistent with previously published metabonomic studies (Huang et al., 2016, 2018; Surendran et al., 2019), the glycerophospholipid metabolism pathway was one of the most critical altered pathways. LysoPC was the most representative differential metabolite. After ischemic necrosis or ischemia-reperfusion injury of cardiomyocytes, LysoPC is hydrolyzed and released into circulation under the action of phospholipase. Serum concentrations are theoretically proportional to the degree of AMI, which explains why, in this study, LysoPC was one of the critical biomarkers of the outcome.

Consistent with our previous findings, the glycerophospholipid metabolism pathway was one of the most critically changed pathways, and LysoPC from the pathway turned out to be the highest weighted differential metabolite. In our previous study, the concentrations of several LysoPCs were significantly downregulated in STEMI compared to PSM healthy controls (Huang et al., 2018). Additionally, a significantly lower concentration of LysoPCs was found in the LMCAD group, i.e., the highest-risk subset in patients with STEMI. In this study, the level of LysoPC (18:0) in patients with UA was higher than in one of the patients with STEMI and lower in DSTEMI than in the SSTEMI group. These findings suggest that the level of this substance is closely related to the severity of myocardial necrosis after STEMI. On the one hand, glycerophospholipids, including LysoPC (18:0), are higher in plasma than serum (Kaluarachchi et al., 2018). Differences are attributed to platelet phospholipase activity during serum clot formation. On the other hand, glycerophospholipids mediate a signaling pathway between monocytes and macrophages (Duong et al., 2004). As a central component of the cell membrane, it also participates in cell proliferation, apoptosis, and energy supply (Borodzicz et al., 2015), all of which are associated with acute myocardial ischemia-reperfusion biological processes. LysoPC (18:0) significantly decreased after the supplementation of medium-chain triglycerides (Xu et al., 2020). Interestingly, we found that the DSTEMI group had a higher plasma level of total cholesterol (TC), with a lower level of LysoPC (18:0), indicating that the excessive dietary intake of TC may lead to poor outcomes.

With the development of metabolomic technology, the level of metabolites *in vivo* can play a diagnostic role in disease or a predictive role in outcome early and can guide personalized treatment to correct metabolic disorders. For example, the levels of LysoPC (18:0) and *N*-acetyl-leukotriene E4 decreased with the development of acute coronary syndrome, and their low levels in the early phase of STEMI morbidity were associated with poorer outcomes in this study. Thus, we infer that raising their levels can retard or reverse the progression. In fact, Buxue Yimu granule, a traditional Chinese medicine, increased the two metabolites in rat plasma (Zhang et al., 2020). The serum levels of LysoPC (18:0) were significantly increased in mice after orally administered Rehmanniae Radix once daily for 10 days (Xia et al., 2020). Fufang Zhenzhu Tiaozhi increased the levels of *N*-acetyl-leukotriene E4 in the livers of mice (Luo et al., 2019). These findings suggest a novel therapeutic strategy to break through the bottleneck of outcome improvement.

Another important finding of this study is that 9cRA, a lipid molecule derived from retinol, is a crucial biomarker of the outcome, second only to LysoPC 18:0 as shown in Figure 5. 9cRA had the best discriminative performance for the LMCAD phenotype in our previous study (Huang et al., 2018). Mechanically, it exerted pleiotropic effects on cellular growth, differentiation, and immune response in all vertebrates by trans-activating several genes (Villamor and Fawzi, 2005). The fact that LMCAD is the highest-risk lesion subset in coronary heart disease and leads to worse outcomes, following STEMI onset can partially explain the findings of this study. Furthermore, 9cRA stimulates spontaneous cortisol secretion and reduces pituitary corticotropin (ACTH) receptor synthesis (Pecori Giraldi et al., 2021). Increased cortisol secretion can lead to sugar, fat, and protein metabolism disorders. The plasma levels of triglyceride and TC in the death group were higher than in the survival group. The causal relationship between them remains to be further demonstrated.

This study has some limitations. First, our sample size was relatively small, partly explained by our strict inclusion criteria, relatively low mortality, and the statistical approach to PSM. Second, our findings should be validated using other STEMI cohorts to provide internal and external validation. Finally, other types of metabonomic platforms or the type of specimen should be applied to validate our findings.

## Conclusion

Using a UPLC/MS platform, we generated a survival prediction model integrating seven metabolites from non-targeted metabonomics and six clinical indicators. This model may be a robust early survival prediction model for patients with STEMI. The validation of both internal and external cohorts is required.

## Data Availability Statement

The original contributions presented in the study are included in the article/Supplementary Material, further inquiries can be directed to the corresponding author.

## Ethics Statement

The studies involving human participants were reviewed and approved by Bioethics Committee of the Third Central Hospital of Tianjin. The patients/participants provided their written informed consent to participate in this study.

## Author Contributions

JL, LH, and SL designed this study and performed the statistical analysis. JL did the data curation for eligible studies. LH plays a major role in the major revision of the manuscript. JL and LH were the major contributors in writing the original draft of the manuscript. XS, CG, and HX contributed to the methodology and the review and editing of the manuscript. All authors read and approved the final manuscript.

## Conflict of Interest

The authors declare that the research was conducted in the absence of any commercial or financial relationships that could be construed as a potential conflict of interest.

## Publisher’s Note

All claims expressed in this article are solely those of the authors and do not necessarily represent those of their affiliated organizations, or those of the publisher, the editors and the reviewers. Any product that may be evaluated in this article, or claim that may be made by its manufacturer, is not guaranteed or endorsed by the publisher.

## Footnotes

1
http://www.genome.jp/kegg/ligand.html

2
http://www.hmdb.ca/
