# Supplementary material for: Clinical Parameters and Metabolomic Biomarkers That Predict Inhospital Outcomes in Patients With ST-Segment Elevated Myocardial Infarctions
Source: Front Physiol. 2022 Feb 8;12:820240. doi: 10.3389/fphys.2021.820240 (PMC8862746; doi:10.3389/fphys.2021.820240)
Supplement: Supplementary file 1 [file Table_1.docx]

Supplemental Table 1. All the metabolites shown in Venn diagram.

| **ID** | Var_38 | Var_40 | Var_323 | Var_107 | Var-22 | Var-310 | Var-214 | Var143 | Var-4 | Var-313 | Var-210 | Var-44 | Var-149 |
| --- | --- | --- | --- | --- | --- | --- | --- | --- | --- | --- | --- | --- | --- |
| **m/z** | 120.08 | 103.05 | 358.37 | 499.29 | 546.35 | 318.24 | 338.27 | 796.53 | 302.30 | 285.13 | 468.31 | 100.08 | 292.10 |
| **RT/min** | 1.16 | 1.16 | 7.94 | 4.31 | 8.59 | 7.29 | 6.31 | 9.95 | 6.74 | 5.77 | 6.73 | 1.17 | 5.20 |
| **Place of Venn diagram** | Ⅰ | Ⅰ | Ⅰ | Ⅰ | Ⅰ | Ⅰ | Ⅰ | Ⅱ | Ⅱ | Ⅱ | Ⅱ | Ⅱ | Ⅱ |
|  |  |  |  |  |  |  |  |  |  |  |  |  |  |
| Var-249 | Var-189 | Var-80 | Var-72 | Var-181 | Var-236 | Var-88 | Var-67 | Var-90 | Var-79 | Var-86 | Var-56 | Var-111 | Var-33 |
| 361.27 | 366.14 | 258.11 | 292.10 | 496.33 | 556.27 | 130.07 | 95.05 | 548.37 | 341.30 | 550.38 | 506.36 | 544.27 | 416.21 |
| 7.02 | 5.64 | 8.60 | 5.22 | 7.45 | 5.72 | 1.97 | 1.16 | 7.85 | 8.58 | 8.80 | 8.60 | 5.71 | 5.71 |
| Ⅱ | Ⅱ | Ⅱ | Ⅱ | Ⅱ | Ⅱ | Ⅲ | Ⅲ | Ⅲ | Ⅲ | Ⅲ | Ⅲ | Ⅳ | Ⅳ |
|  |  |  |  |  |  |  |  |  |  |  |  |  |  |
| **ID** | Var-58 | Var-24 | Var-82 | Var-6 | Var-329 | Var-59 | Var-263 | Var-34 | Var-250 | Var-265 | Var-272 | Var-30 | Var-312 |
| **m/z** | 502.33 | 483.25 | 558.29 | 520.34 | 518.32 | 316.32 | 311.29 | 546.35 | 161.02 | 645.32 | 560.31 | 485.26 | 168.07 |
| **RT/min** | 7.12 | 7.14 | 7.16 | 7.12 | 6.03 | 7.03 | 8.50 | 7.36 | 1.32 | 5.71 | 7.68 | 7.67 | 5.26 |
| **Place of Venn diagram** | Ⅳ | Ⅳ | Ⅳ | Ⅳ | Ⅴ | Ⅴ | Ⅴ | Ⅴ | Ⅴ | Ⅴ | Ⅴ | Ⅴ | Ⅴ |
|  |  |  |  |  |  |  |  |  |  |  |  |  |  |
| Var-197 | Var-262 | Var-1 | Var-41 | Var-289 | Var-27 | Var-237 | Var-253 | Var-19 | Var-330 | Var-99 | Var-106 | Var-39 | Var-85 |
| 991.67 | 274.09 | 496.34 | 288.29 | 287.14 | 568.34 | 283.26 | 487.29 | 340.36 | 519.31 | 702.34 | 398.20 | 480.34 | 562.33 |
| 7.48 | 4.04 | 7.47 | 6.46 | 5.73 | 7.03 | 7.90 | 8.51 | 7.92 | 5.83 | 5.71 | 5.71 | 7.80 | 8.60 |
| Ⅴ | Ⅴ | Ⅴ | Ⅴ | Ⅴ | Ⅴ | Ⅴ | Ⅴ | Ⅴ | Ⅵ | Ⅵ | Ⅵ | Ⅵ | Ⅵ |
|  |  |  |  |  |  |  |  |  |  |  |  |  |  |
| **ID** | Var-110 | Var-309 | Var-172 | Var-316 | Var-234 | Var-251 | Var-93 | Var-74 | Var-96 | Var-62 | Var-117 | Var-112 |  |
| **m/z** | 217.08 | 481.26 | 482.32 | 563.41 | 232.99 | 331.21 | 130.05 | 532.34 | 361.27 | 504.34 | 169.10 | 129.10 |  |
| **RT/min** | 5.51 | 4.37 | 7.06 | 9.90 | 5.60 | 5.05 | 5.70 | 7.95 | 7.01 | 7.67 | 5.70 | 5.40 |  |
| **Place of Venn diagram** | Ⅵ | Ⅵ | Ⅶ | Ⅶ | Ⅶ | Ⅶ | Ⅶ | Ⅶ | Ⅶ | Ⅶ | Ⅶ | Ⅶ |  |

Abbreviations: RT= Retention Time;
